# Vitamin D status associates with skeletal muscle loss after anterior cruciate ligament reconstruction

**DOI:** 10.1172/jci.insight.170518

**Published:** 2023-12-08

**Authors:** Yuan Wen, Christine M. Latham, Angelique N. Moore, Nicholas T. Thomas, Brooke D. Lancaster, Kelsey A. Reeves, Alexander R. Keeble, Christopher S. Fry, Darren L. Johnson, Katherine L. Thompson, Brian Noehren, Jean L. Fry

**Affiliations:** 1 Center for Muscle Biology, College of Health Sciences,; 2 Department of Physiology, College of Medicine,; 3 Division of Biomedical Informatics, Department of Internal Medicine, College of Medicine,; 4 Department of Orthopaedic Surgery & Sports Medicine, and; 5 Dr. Bing Zhang Department of Statistics, University of Kentucky, Lexington, Kentucky, USA.

**Keywords:** Muscle Biology, Bioinformatics, Orthopedics, Skeletal muscle

## Abstract

**BACKGROUND:**

Although 25-hydroxyvitamin D [25(OH)D] concentrations of 30 ng/mL or higher are known to reduce injury risk and boost strength, the influence on anterior cruciate ligament reconstruction (ACLR) outcomes remains unexamined. This study aimed to define the vitamin D signaling response to ACLR, assess the relationship between vitamin D status and muscle fiber cross-sectional area (CSA) and bone density outcomes, and discover vitamin D receptor (VDR) targets after ACLR.

**METHODS:**

Twenty-one young, healthy, physically active participants with recent ACL tears were enrolled (17.8 ± 3.2 years, BMI 26.0 ± 3.5 kg/m^2^). Data were collected through blood samples, vastus lateralis biopsies, dual energy x-ray bone density measurements, and isokinetic dynamometer measures at baseline, 1 week, 4 months, and 6 months after ACLR. The biopsies facilitated CSA, Western blotting, RNA-seq, and VDR ChIP-seq analyses.

**RESULTS:**

ACLR surgery led to decreased circulating bioactive vitamin D and increased VDR and activating enzyme expression in skeletal muscle 1 week after ACLR. Participants with less than 30 ng/mL 25(OH)D levels (*n* = 13) displayed more significant quadriceps fiber CSA loss 1 week and 4 months after ACLR than those with 30 ng/mL or higher (*n* = 8; *P* < 0.01 for post hoc comparisons; *P* = 0.041 for time × vitamin D status interaction). RNA-seq and ChIP-seq data integration revealed genes associated with energy metabolism and skeletal muscle recovery, potentially mediating the impact of vitamin D status on ACLR recovery. No difference in bone mineral density losses between groups was observed.

**CONCLUSION:**

Correcting vitamin D status prior to ACLR may aid in preserving skeletal muscle during recovery.

**FUNDING:**

NIH grants R01AR072061, R01AR071398-04S1, and K99AR081367.

## Graphical abstract

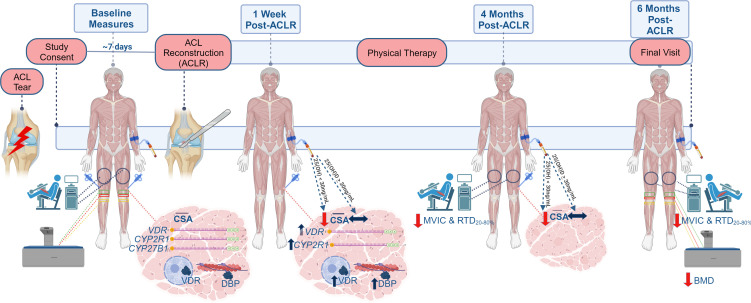

## Introduction

The anterior cruciate ligament (ACL) is frequently susceptible to injury, constituting a majority of noncontact knee injuries. It affects over 200,000 individuals annually in the United States alone (1), predominantly physically active adolescents (2). The preferred treatment for patients experiencing persistent knee laxity is ACL reconstruction (ACLR). However, the procedure does not entirely alleviate long-term deficits in lower limb structure and function. After ACLR, patients often exhibit a 20%–40% reduction in quadriceps strength for several years (3), which associates with ongoing functional impairment lasting for at least 3 years after ACLR (4, 5).

Additionally, there is a notable decrease in bone mineral density (BMD) in the proximal tibia and distal femur within the first 6 months after ACLR (6, 7). Significant reductions in the distal femur BMD persist for at least 2 years (7). These deficits in strength and BMD substantially heighten the risk of knee osteoarthritis development (8, 9). Many potential causes of long-term deficits have been scrutinized, but there remains a pressing need for clinical interventions that can reliably improve patient outcomes (10). Consequently, in-depth understanding of the treatment options and long-term consequences may substantially enhance patients’ health span.

The impact of vitamin D status on ACLR outcomes remains largely unknown. Vitamin D, an endogenously synthesized steroid hormone and dietary component, is unequivocally essential for proper bone mineralization in youth (11). Vitamin D functions primarily through hormone activity by binding to the its nuclear receptor (VDR) and influencing expression of approximately 2000 human genes (12). Circulating 25-hydroxyvitamin D [25(OH)D] concentrations of 30 ng/mL or higher (75 nmol/L), surpassing the US National Academy of Medicine established deficiency cutoff points (11), are associated with reduced risk of stress fractures and sports injuries (13, 14), greater grip strength (15), and a range of other health outcomes (16–18). Studies show that participants with higher vitamin D status exhibit enhanced muscle strength and recovery (19, 20). Moreover, vitamin D supplementation may boost lower body strength in athletes (21). In preclinical models, overexpression of VDR causes muscle hypertrophy (22), while VDR knockdown induces muscle atrophy (23). Chemical injury in rodent skeletal muscle promotes expression of VDR and cytochrome P450 2R1 (CYP27B1, a vitamin D–activating enzyme) (24). However, despite the apparent benefits of vitamin D’s bolstering of skeletal muscle health, VDR presence in healthy, mature human skeletal muscle tissue is nearly undetectable (25, 26).

**Figure 1:**
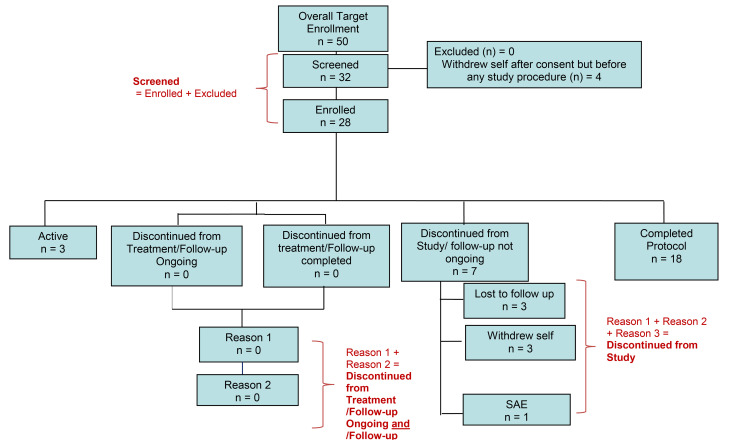
STROBE diagram for parent study enrollment at time of data analysis. This study includes data from participants in the “Active” and “Completed” groups (*n* = 21).

ACL tears and subsequent ACLR often lead to persistent muscle weakness (27), and effective therapies to entirely mitigate the muscle and strength loss with these conditions are yet to be identified. Here we sought to characterize vitamin D–associated activity in quadriceps muscle after ACLR, identify potential VDR gene targets after ACLR, and determine how vitamin D status associates with skeletal muscle size, BMD, and strength outcomes using samples from an ongoing observational study (Figure 1). We aimed to examine whether ACLR would stimulate the expression of genes within vitamin D–related pathways through the transcriptional activation activities of VDR at the chromosomal level. This was achieved through a multiomic integration approach utilizing bulk RNA sequencing (RNA-seq) of skeletal muscle and chromatin immunoprecipitation combined with sequencing (ChIP-seq) of VDR. We hypothesized that optimal vitamin D status [25(OH)D ≥ 30 ng/mL] would associate with reduced loss of skeletal muscle size and femoral BMD in the injured (operative) limb. Accordingly, we set our co-primary outcomes as quadriceps skeletal muscle fiber cross-sectional area (CSA) and BMD, respectively.

## Results

### Circulating biomarkers.

**Figure 2:**
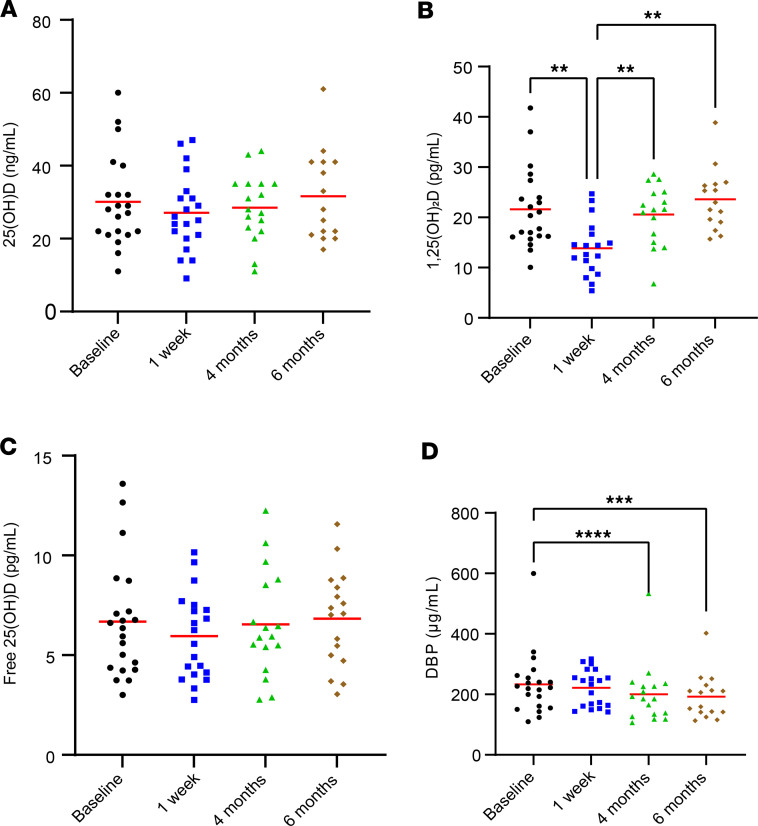
Circulating vitamin D metabolites before and after ACL reconstruction (ACLR). (**A**) 25-Hydroxyvitamin D [25(OH)D; status indicator] was unchanged throughout the study (*P* = 0.360). (**B**) 1,25-Dihydroxyvitamin D [1,25(OH)_2_D; active form] was significantly reduced after ACLR (*P* = 0.002). (**C**) Free 25(OH)D was unchanged throughout the study (*P* = 0.433). (**D**) Vitamin D binding protein (DBP) was lower at 4- and 6-month follow-ups when compared with baseline and 1 week after ACLR (*P* ≤ 0.0001). Participants: *n* = 21, 20, 17, and 17, for baseline, 1-week, 4-month, and 6-month measures, respectively. All available samples were analyzed. One-way repeated-measures ANOVA; results of post hoc tests on graph. **P < 0.01; ***P < 0.001; ****P = 0.0001.

Relative to baseline measures, a substantial reduction in 1,25-dihydroxyvitamin D [1,25(OH)_2_D] was observed 1 week after ACLR (22 ± 8 pg/mL vs. 14 ± 5 pg/mL; *P* = 0.0041 for baseline vs. 1 week; *P* = 0.0024 main effect for time). Vitamin D binding protein (DBP) showed a significant decrease at 4- and 6-month follow-ups when compared with baseline (233.2 ± 103.4 μg/mL vs. 200.5 ± 99.2 μg/mL and 192 ± 73.6 μg/mL; *P* < 0.001 for main effect). However, there was no significant change in circulating total and free 25(OH)D throughout the duration of the study. The findings of circulating biomarkers are graphically represented in Figure 2. Comprehensive time-course data for all participants are available in Supplemental Table 1; supplemental material available online with this article; https://doi.org/10.1172/jci.insight.170518DS1, and data groups by vitamin D status are provided in Supplemental Table 2.

### Enhanced expression of vitamin D–linked genes and proteins after ACLR.

**Figure 3:**
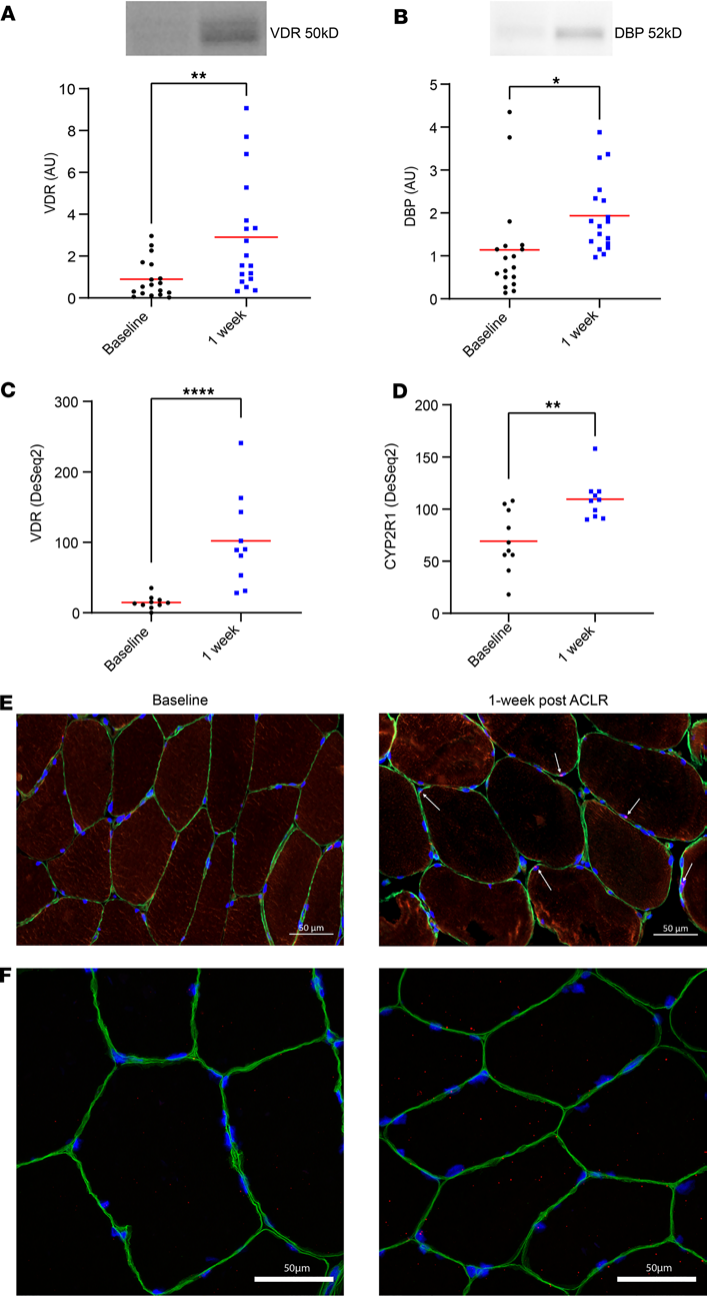
Vitamin D–associated transcripts and proteins in vastus lateralis were elevated in response to ACL reconstruction (ACLR). (**A**) Vitamin D receptor (VDR; protein AU) on Western blot increased in response to ACLR (*n* = 18; *P* = 0.003). (**B**) Vitamin D binding protein (DBP) as indicated by Western blots increased from baseline in response to ACLR (*P* = 0.02). (**C**) Vitamin D receptor (VDR) RNA-seq transcript count increased from baseline in response to ACLR (*n* = 10). (**D**) Cytochrome P450 2R1 (*CYP2R1*) RNA-seq transcript count increased in response to ACLR. (**E**) Representative image of VDR protein in IHC analysis of the injured limb at baseline and 1 week after ACLR (1 participant from experiment reported in **A**). (**F**) RNAscope in situ hybridization completed to visualize VDR mRNA in the quadriceps of the injured limb at study baseline and 1 week after ACLR. RNAscope was completed on 1 participant showing substantially increased VDR on RNA-seq. Scale bars: 50 μm. Images of individual channels are included in Supplemental Figure 2. One-way repeated-measures ANOVA; results of post hoc tests on graph. *P < 0.05; **P < 0.01; ****P < 0.0001. Adjusted *P* values are presented in **C** and **D**.

Significant increases were observed in the quadriceps of the injured limb after ACLR for protein abundances of both VDR (0.90 ± 0.92 AU vs. 2.91 ± 2.67 AU; *P* = 0.003) and DBP (1.14 ± 1.15 AU vs. 1.93 ± 0.86; *P* = 0.02). Bulk RNA-seq data were queried for potential changes in vitamin D–associated pathways, including *VDR*, GO:0042368 — vitamin D biosynthetic process (*CYP27B1* and *CYP2R1*), GO:0042369 — vitamin D catabolic process (*CYP3A4*, *FGF23*, and *CYP24A1*), GO:0070640 — vitamin D3 metabolic process (*UTG1A3* and *UGT1A4*), and additional genes in the parent class GO:0042359 — vitamin D metabolic process (*LRP2*, *CYP11A1*, and *GC*). RNA-seq data demonstrated a significant upregulation in the expression of *VDR* (adjusted *P* < 0.05) and *CYP2R1* (adjusted *P* < 0.05) at the 1 week mark when compared with baseline for both injured and healthy limbs. *CYP3A4*, *CYP27B1*, and *CYP11A1* did not show significant changes. *FGF23*, *CYP24A1*, *UTG1A3*, *UGT1A4*, *LRP2*, and *GC* were not detected or were very low in abundance (>80% participants showing no transcripts). No significant changes were detected in expression of several genes in GO:0010957 — negative regulation of vitamin D biosynthetic process, including *GFI1*, *NFKB1*, *SNAI1*, and *SNAI2*. Taken together, the data suggest a selective transport of vitamin D metabolites into the skeletal muscle following ACLR, as well as increased VDR expression and conversion of vitamin D to 25(OH)D. Comprehensive data can be found in the supplemental Supporting Data Values Excel file, and data from the injured limb is illustrated in Figure 3.

### VDR targets genes associated with muscle structure and energy generation genes after ACLR.

VDR exerts its primary role as a nuclear transcription factor in the presence of vitamin D’s active form, 1,25(OH)_2_D. To ascertain the genomic locations where VDR binds in skeletal muscle, we conducted ChIP-seq analysis on pooled muscle biopsies from 1 week after ACLR, a time point marked by significant VDR protein elevation. We identified a total of 3290 peaks, 219 of which were strong peaks in proximity to tRNAs. The significance of this finding is unclear, but due to concerns that such hyper-intense signals are technical artifacts of ChIP-seq (28), these peaks were excluded from subsequent analysis. Detailed results are provided in the Supporting Data Values file.

**Figure 4:**
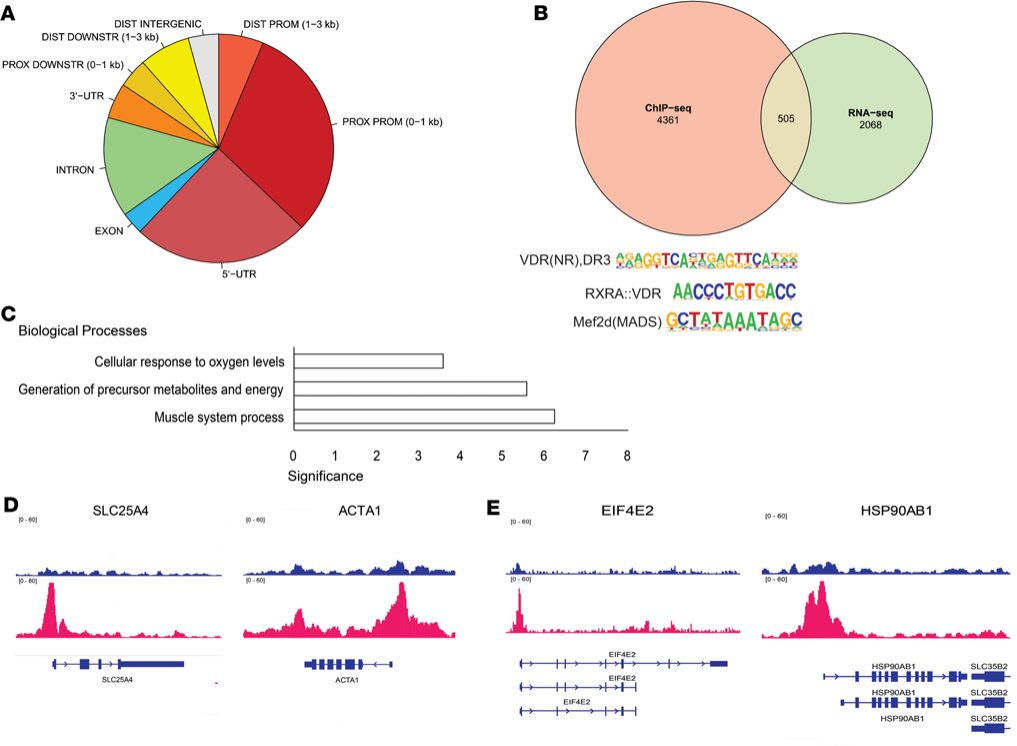
VDR targets genes associated with muscle structure and energy generation genes after ACL reconstruction (ACLR). (**A**) Distribution of ChIP-seq peaks’ relative gene sequences, including untranslated regions (UTRs), proximal (PROX) and distal (DIST) promoters (PROM), and regions downstream of the 3′-UTR (DOWNSTR). (**B**) Overlap between genes with significant VDR binding that were also differentially expressed 1 week after ACLR, along with enriched VDR binding sequence motifs. (**C**) Gene ontology analysis of common 505 genes and their significantly enriched biological processes. (**D**) ChIP-seq reads for input (blue, top) and VDR (red) showing peaks for ATP transporter, *SLC25A4*, and skeletal α-actin, *ACTA1*. Coding sequence for each gene is shown in blue below the VDR red peaks. Rectangles are exons and the arrows along the introns indicate direction of mRNA transcription. (**E**) ChIP-seq reads for input (blue, top) and VDR (red) showing peaks at the promoters of eukaryotic initiation factor, *EIF4E2*, and heat shock protein, *HSP90AB1*. Coding sequences (blue) are shown for multiple splice isoforms.

The locations of the detected peaks’ relative transcription start sites (TSSs) are shown in Figure 4A. The majority of detected peaks localized to proximal promoters 0–1 kb upstream of the TSS and the 5′-UTR, followed by introns and then regions 1–3 kb downstream of the transcript termination site. Significantly, 75.6% of all peaks were within 200 bp of CpG islands compared with the estimated 1.8% in the random control, suggesting a specificity toward genomic regulatory elements. Motif enrichment analysis confirmed the presence of 3 motifs, including VDR:D3 (29), VDR:RXRA, and Mef2d (Figure 4B). A total of 4866 genes were associated with detected VDR binding peaks, and 505 of these overlapped with the 2573 significantly differentially expressed genes (DEGs) identified using RNA-seq analysis. Gene ontology analysis revealed that these overlapping genes are significantly enriched in biological processes involved in muscle-specific gene expression, metabolite and energy generation, and cellular oxygen levels (Figure 4C). We found significant VDR binding to the promoter of *SLC25A4* (Figure 4D), a crucial gene for energy metabolism responsible for transport of ATP out of the mitochondria and into the cytoplasm (30). VDR also bound to the gene promoter of a key muscle cytoskeletal protein, skeletal α-actin (*ACTA1*) (Figure 4D). Our data showed a significant decrease in the expression of *SLC25A4* and *ACTA1*, suggesting that VDR may enhance these genes’ transcription to counteract the loss of gene expression after ACLR. VDR binding was also detected at the promoters of genes involved in protein synthesis, namely *EIF4E2* and *HSP90AB1* (Figure 4E). These genes both showed increased transcript abundance in the RNA-seq analysis.

### Multiomic integration of ChIP-seq ond RNA-seq data.

**Figure 5:**
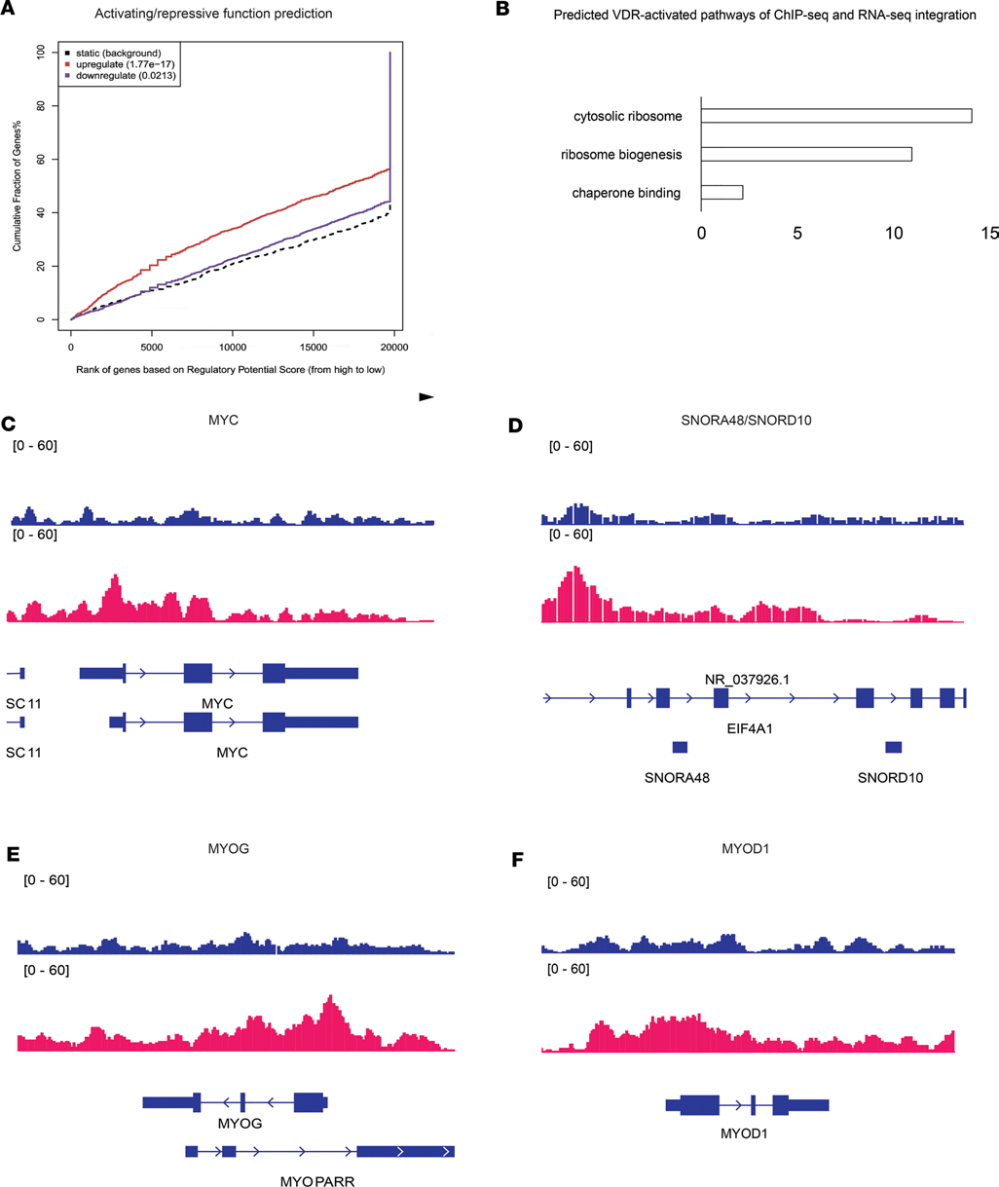
Multiomic integration of ChIP-seq and RNA-seq data implicates the role of VDR in regulating muscle ribosome biogenesis. (**A**) ChIP-seq peaks and RNA-seq differentially expressed genes (DEGs) were integrated by calculating an activation and an inhibition score to estimate the effects of transcriptional regulation. Genes are ranked by score and VDR binding is associated with highly significant activation (red solid line) of a subset of DEGs relative to background (black dotted line). The small subset of DEGs showing significant inactivation by VDR binding is represented by the blue solid line. (**B**) Top 3 enriched biological processes for VDR-activated DEGs. ChIP-seq reads for input (blue, top) and VDR (red) showing VDR binding at the genomic locations for MYC (**C**), SNORA4B/SNORD10 (**D**), MYOG (**E**), and MYOD1 (**F**). Coding sequence for each gene is shown in blue below the VDR red peaks. Rectangles are exons and arrows along the introns indicate direction of mRNA transcription. Multiple splice isoforms are shown.

To delve deeper into the role of VDR binding activity in muscle after ACLR, we performed a multiomic integration analysis using BETA (31). The regulatory potential score for each peak predicted that VDR is highly activating (*P* = 1.77 × 10^–17^) and does not show a significant repressive function (Figure 5A). A total of 841 genes were significantly regulated by VDR in muscle after ACLR. Gene ontology analysis revealed that these genes were highly significantly enriched for components of the ribosome and biogenesis of the cellular translation machinery (Figure 5B). VDR peaks were found in transcription factors like *MYC* (Figure 5C), which has a potent activating effect on new ribosome production and is known to enhance rRNA transcription. Numerous small nucleolar RNAs (snoRNAs) were also significantly enriched for VDR binding peaks (Figure 5D), suggesting a coordinated process to increase both rRNA synthesis and maturation. Furthermore, key muscle-specific transcription factors, *MYOG* (Figure 5E) and to a lesser extent *MYOD1* (Figure 5F), also showed enrichment for VDR binding at the proximal promoter region.

### Low vitamin D status associates with fiber CSA loss 1 week and 4 months after ACLR.

Status groups showed no significant differences in CSA of the injured limb at baseline (4455 ± 849 μm^2^ vs. 4291 ± 1046 μm^2^; *P* = 0.391). Among participants with an average total 25(OH)D of less than 30 ng/mL, CSA was lower 1 week and 4 months after ACLR (4455 ± 849 μm^2^ vs. 3285 ± 717 μm^2^ and 3119 ± 418 μm^2^, respectively; *P* < 0.01 for post hoc comparisons; *P* = 0.041 for time × vitamin D status interaction). In contrast, no significant decreases were observed among those with 25(OH)D of 30 ng/mL or higher (4291 ± 1046 μm^2^ vs. 4112 ± 1364 μm^2^ and 3867 ± 615 μm^2^, respectively; *P* > 0.05 for all post hoc comparisons).

**Figure 6:**
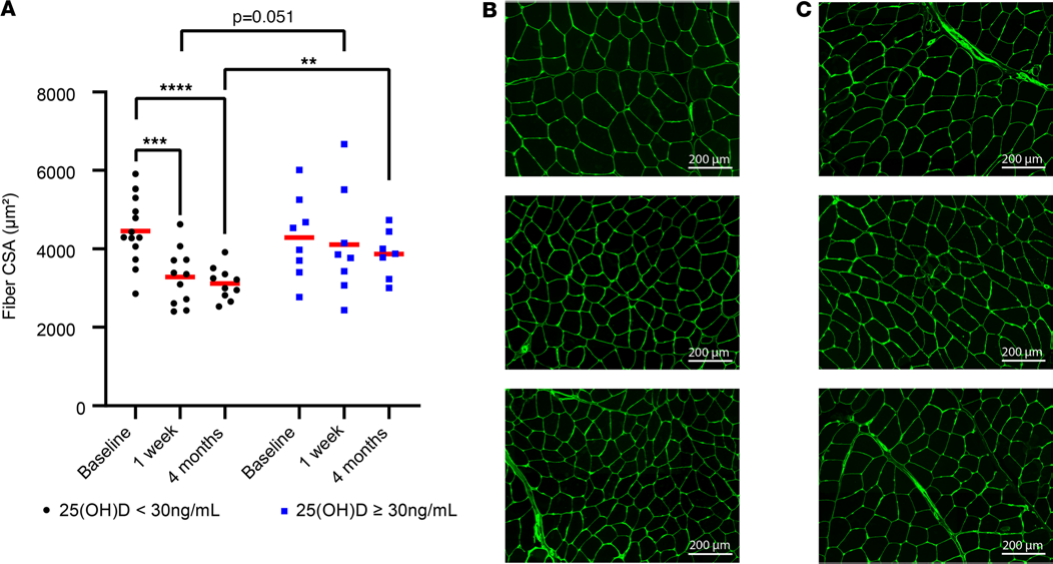
Mean study 25(OH)D less than 30 ng/mL associates with cross-sectional area (CSA) reductions in the vastus lateralis muscle. (**A**) When comparing participants with low study vitamin D status [25(OH)D < 30 ng/mL] and those with adequate status [25(OH)D ≥ 30 ng/mL], the low vitamin D status group showed significant reductions in fiber CSA at 1-week and 4-month follow-ups when compared with baseline. At 4 months, the fiber CSA was significantly lower in participants having study 25(OH)D < 30 ng/mL (*n* = 10) when compared with participants who had 25(OH)D ≥ 30 ng/mL (*n* = 7). (**B**) Representative CSA IHC for a low-status participant. (**C**) Representative CSA IHC for an adequate-status participant. **B** and **C** are representative images for the experiment shown in **A** having 21 total participants at the baseline and 1-week time points and 17 participants at the 4-month mark. Scale bars: 200 μm. Full-factorial repeated-measures ANOVA with post hoc tests. The model showed an overall time × vitamin D status interaction effect *P* = 0.041; results of host-hoc tests on graph. **P < 0.01; ***P < 0.001; ****P < 0.0001.

At the 4-month follow-up, post hoc analyses show CSA values were lower in the injured limb of participants with total 25(OH)D of less than 30 ng/mL compared with the 4-month CSA values of those with 25(OH)D of 30 ng/mL or higher (3119 ± 418 μm^2^ vs. 3867 ± 615 μm^2^; *P* < 0.01). CSA trended lower in the injured limb of participants with total 25(OH)D of less than 30 ng/mL when compared with those with 25(OH)D of 30ng/mL or higher at 1 week after ACLR (3285 ± 717 μm^2^ vs. 4112 ± 1364 μm^2^; *P* = 0.051). Figure 6A shows CSA by vitamin D status group, Figure 6, B and C show representative CSA IHC images for participants with both low and high at baseline, 1 week after ACLR and 4 months after ACLR, respectively. Supplemental Figure 3 shows a comparable graph displaying minimum Feret diameter (MFD) by status group. Morphological assessment of quadriceps muscle fibers was also performed with H&E staining, and we did not observe overt signs of muscle damage in either vitamin D status group (Supplemental Figure 4). The complete data are provided in Supplemental Tables 3 and 4.

### Vitamin D status does not associate with strength, power, or bone density outcomes.

**Figure 7:**
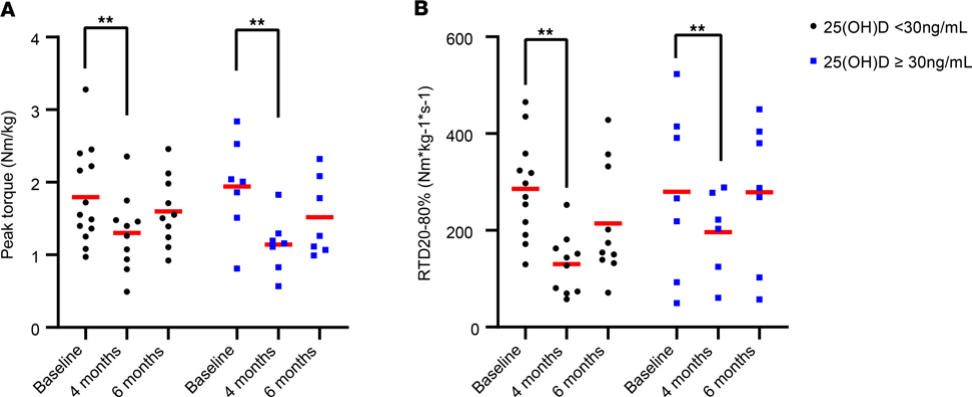
Study 25(OH)D does not associate with reductions in strength or power. (**A**) There were no differences in weight-normalized peak torque in participants with study 25(OH)D < 30 ng/mL (*n* = 13, 10, and 10 at baseline, 1 week, and 4 months, respectively) when compared with participants having study 25(OH)D ≥ 30ng/mL (*n* = 8, 7, and 7 at baseline, 1 week, and 4 months, respectively). (**B**) Participants lost significant power over time, but there were no differences between status groups. The power measure, RTD_20%–80%_, refers to the mean slope of the torque-time curve between 20% and 80% of the first 200 milliseconds from muscle contraction onset. Full-factorial repeated-measures ANOVA. **P < 0.01 for overall effect of time.

**Figure 8:**
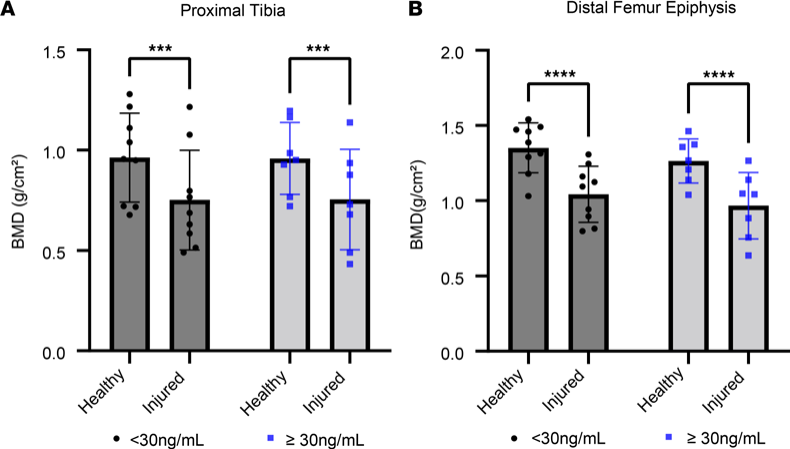
Comparable bone mineral density (BMD) loss in participants with study 25(OH)D less than 30 ng/mL and 25(OH)D less than 30 ng/mL. The figure shows BMD from the final 6-month DXA scan from the injured (surgical) and healthy (nonsurgical) limbs. (**A**) BMD in proximal tibia. (**B**) BMD in distal femur. All participants showed lower BMD in the injured limb at the 6-month follow-up and there were no differences between groups (*n* = 9 and 7 for low- and high-status groups, respectively). Full-factorial repeated-measures ANOVA; results of post hoc tests on graph. ***P < 0.001, ****P < 0.0001.

For all 3 BMD regions assessed, normalized peak torque, and RTD_20%–80%_, there was a significant main effect of time, indicating an average decrease in all values over time. However, none of these indicators showed differences between participants with an average total 25(OH)D of less than 30 ng/mL compared with those with a total 25(OH)D of 30 ng/mL or higher (*P* > 0.05 for status × time interactions). Data are presented in Supplemental Tables 5–8, and Figure 7 and Figure 8.

### Identification of genes responsive to vitamin D status after ACLR.

**Figure 9:**
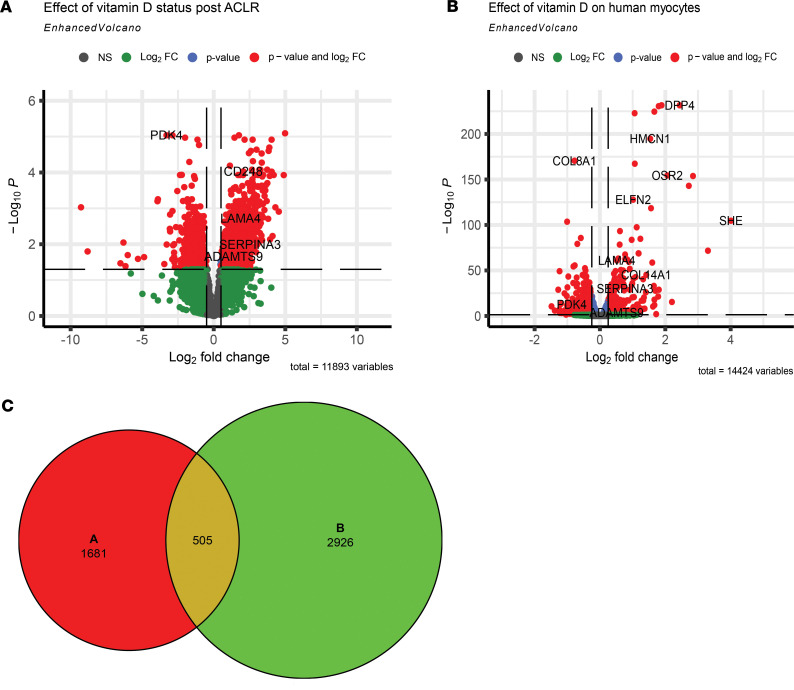
Identification of genes responsive to vitamin D status after ACL reconstruction (ACLR). (**A**) Volcano plots of DEGs from analysis of RNA-seq data sets comparing low and high vitamin D status groups 1 week after ACLR log_2_-transformed fold change (Log2FC) cutoff was set at ±1 (2-fold up and down). Green represents genes that did not reach statistical significance (adjusted *P* < 0.05). Blue represents genes that were statistically significantly different but did not change more than the fold change cutoff. Red represents DEGs meeting both statistical and fold change cutoffs. (**B**) Vehicle- and 1,25(OH)_2_D3-treated human primary myocytes. (**C**) Venn diagram showing the DEGs from **B** and **C**, with 505 common to both.

Given the observed association between low vitamin D status and decrease in fiber CSA, we sought to identify genes that were most responsive after ACLR by vitamin D status. We identified 2186 DEGs (adjusted *P* < 0.05) between high and low vitamin D status after ACLR using our RNA-seq data. Additionally, using a publicly available RNA-seq data set from skeletal myocytes treated with 1,25(OH)_2_D in vitro (32), we found 3431 DEGs. Volcano plots for both data sets are shown in Figure 9, A and B, respectively. To pinpoint genes more directly related to vitamin D status, we compared these 2 data sets and found 505 common genes, with 143 showing changes in the in the same direction (Figure 9C). A selection of the notable top increased DEGs in both data sets include collagen (*COL14A1*), laminin (*LAMA4*), protease inhibitor (*SERPINA3*), and metalloprotease (*ADAMTS9*), which are all vital for cellular remodeling. Additionally, among the increased genes were *ITGA6* and *CD248*, both of which play a role in signaling muscle stem cell differentiation and angiogenesis.

**Figure 10:**
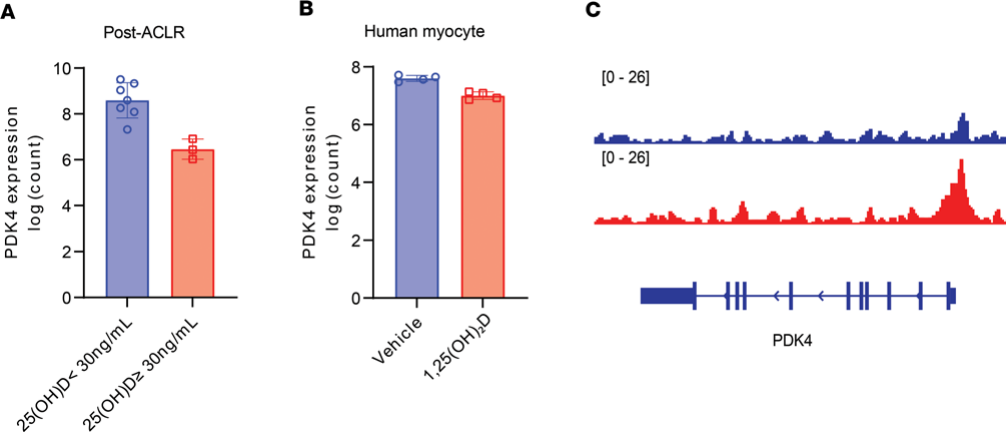
Identification of genes responsive to vitamin D status after ACL reconstruction. (**A**) Comparison of change in *PDK4* transcript counts in vastus lateralis of the injured limb in participants above and below the 30 ng/mL vitamin D status cutoff point (*n* = 10). (**B**) *PDK4* transcript fold change in vehicle- and 1,25(OH)_2_D3-treated human primary myocytes (*n* = 4 per group). (**C**) Coding sequence for PDK4 is shown in blue below the VDR red peaks. Rectangles are exons and arrows along the introns indicate direction of mRNA transcription. Multiple splice isoforms are shown. Adjusted *P* values presented in **A** and **B**.

Notably, one of the most downregulated genes in both data sets is *PDK4* (Figure 10, A and B), which is the master regulator of muscle metabolism and inhibits the pyruvate dehydrogenase complex. By downregulating *PDK4*, vitamin D likely promotes pyruvate conversion to acetyl-CoA and may promote a shift toward glucose as a fuel source, as previously reported (32, 33). Although VDR binding at the *PDK4* promoter did not reach statistical significance (Figure 10C), regulation may have occurred at an earlier time point than we measured to influence *PDK4* transcription. *COL14A1* was the only target common to all data sets.

## Discussion

Our findings illustrate that participants with higher levels of 25(OH)D (≥30 ng/mL) experienced a smaller reduction in the co-primary endpoint of quadriceps fiber CSA at the 1-week and 4-month postsurgery checkpoints following ACLR. Surgical reconstruction of the ACL triggered an acute increase in the expression of *VDR* and vitamin D biosynthetic enzymes in the quadriceps muscle. However, participants with 25(OH)D concentrations of 30 ng/mL or higher exhibited a comparable degree of femoral BMD loss (co-primary endpoint) as their counterparts with lower vitamin D levels. These findings underscore the crucial role of vitamin D availability and status as vital nutritional considerations in the perioperative and immediate postoperative phases of ACLR.

Previous research has demonstrated that circulating vitamin D metabolites are negative acute-phase reactants. In one study investigating patients receiving elective knee or hip surgery, 25(OH)D was significantly reduced 2 days after surgery (34). Others found that elective hip replacement surgery also promotes reductions in 1,25(OH)_2_D that are detectable several weeks after the procedure (35). While it remains uncertain whether the decrease in 1,25(OH)_2_D observed after ACLR is due to reduced synthesis, increased catabolism, or greater uptake, our finding of elevated DBP protein in skeletal muscle following ACLR suggests that the surgery may stimulate greater tissue uptake of vitamin D metabolites.

To identify chromosomal targets of VDR binding, we expected and were able to detect known VDR motifs (29, 36, 37). Unexpectedly, the ChIP-seq analysis showed significant enrichment of a known MEF2D binding motif. The MEF2 family of transcription factors is muscle specific and interacts with muscle lineage determination factors such as MYOD1 and MYOG (38, 39). The implication of this finding is unclear, but VDR association with tissue-specific transcription activation complexes, either directly or indirectly, could represent a potential mechanism through which vitamin D and VDR affect muscle structure and function. There were significant VDR binding peaks at the loci for muscle lineage–specific regulatory factors, *MYOG* and *MYOD1*, as well as muscle-specific cytoskeletal protein, *ACTA1*, further highlighting the complex interaction of VDR with the cellular regulators to mediate tissue-specific adaptations. Collectively, these findings suggest an underappreciated role of VDR in skeletal muscle response to recovery from injury.

Additionally, our findings suggest that VDR target genes may regulate protein synthesis in skeletal muscle by modulating protein translation capacity. We found VDR binding not only to the promoters’ initiation factors, but also to those of heat shock proteins. Furthermore, MYC, as a potent driver of ribosome biogenesis, plays a significant role in protein synthesis in skeletal muscle tissue. The discovery of VDR binding at this particular locus bolsters the argument for VDR’s role in enhancing muscle capacity for protein synthesis (40–42). We also noted significant VDR peaks at the promoters of multiple snoRNAs, which are necessary regulators in the maturation of ribosomal RNA (43, 44). Protein synthesis usually shows an inverse relationship between speed and fidelity (45). It is possible that VDR increases the speed of protein production while also ensuring proper protein folding and translational fidelity by promoting expression of snoRNAs, while concurrently increasing ribosome efficiency and total synthetic capacity.

Our study revealed that participants with high vitamin D status exhibited markedly different muscle transcriptomic signatures compared with those with low status. By leveraging existing high-resolution transcriptomics data sets, we identified a significant association between vitamin D status and *PDK4*, a key regulator of metabolism via its inhibitory action on pyruvate dehydrogenase. Although we did not establish a direct mechanism through which VDR regulates *PDK4* expression, this finding suggests that vitamin D may regulate muscle cell metabolism after ACLR by supporting glucose metabolism. Given our findings that VDR regulates genes integral for skeletal muscle recovery, we advocate for the early correction of low vitamin D status as an actionable intervention that could improve quadriceps muscle energetics and translational capacity after ACLR.

### Vitamin D status is not associated with BMD loss.

We did not observe any relationship between vitamin D status defined with 25(OH)D and loss of BMD, and both groups experienced substantial loss of BMD in the proximal tibia and distal femur. Cross-sectional studies have typically failed to demonstrate a strong link between circulating total 25(OH)D and BMD (46, 47), and regular physical activity is a key determinant of BMD in adolescents (48). Although severe vitamin D deficiency undeniably impairs bone mineralization (11), it appears that marginal vitamin D status is not a primary driver of BMD loss in the injured leg after ACLR.

### Vitamin D status is not associated with decrements in strength and power.

In our study, participants with 25(OH)D of 30 ng/mL or higher did not show better maintenance of normalized peak torque or RTD_20%–80%_ after ACLR when compared to those with concentrations of less than 30 ng/mL. Some studies supplementing athletes with vitamin D have not demonstrated efficacy in improving strength or functional outcomes (49). For instance, in one trial, adolescent swimmers with 25(OH)D of less than 30 ng/mL took vitamin D drops providing 2000 IU/day for 12 weeks with the goal of reaching 30 ng/mL. Despite significant increases in total circulating 25(OH)D and a 9.3 ng/mL difference between study groups at the trial’s conclusion, vitamin D supplementation did not increase grip strength or promote better performance on balance and swim tests. Nonetheless, a meta-analysis of the effect of vitamin D supplementation on power, strength, and muscle mass showed small increases in muscle strength with vitamin D supplementation, but no increases in power or muscle mass (19). At the same time, older people with a vitamin D concentration of less than 12 ng/mL show more substantial strength gains with vitamin D supplementation (19). Gupta and others (50) showed that following ACLR, patients with 25(OH)D of less than 20 ng/mL had a graft failure rate of approximately 6% compared with a rate of 2% in patients with a concentration of 30 ng/mL; however, these outcomes were not statistically significant. Based on data from the Multicenter Orthopedic Outcomes Network (MOON) cohort, factors such as high body mass index, smoking, subsequent knee surgeries, and severe medial, lateral, and patellofemoral cartilage lesions are predictive of functional outcomes 10 years after ALCR (51). However, the long-term relationship between diet and ACL outcomes remains largely unexplored.

### Clinical implications.

Vitamin D clinical cutoff points were originally established to prevent and treat frank vitamin D deficiency diseases like rickets and osteomalacia (11). However, these same cutoff points may not be adequate for optimizing health and facilitating recovery from injuries. While our study does not provide sufficient evidence to conclude that increasing vitamin D concentrations to 30 ng/mL will improve clinical outcomes in ACLR patients, this target is widely accepted as sufficient without being excessive. The Endocrine Society Clinical Practice Guidelines broadly support an optimization cutoff point of 30 ng/mL and indicates that children with concentrations of less than 20 ng/mL may reach 30 ng/mL by supplementing with 2000 IU/day (50 μg/day) for 1 year (52). For adults, supplementation with 50,000 IU weekly (or 6000 IU daily) for 8 weeks can help achieve concentration of 30 ng/mL. In the long term, vitamin D supplementation should not exceed 4000 IU/day to avoid toxicity (11, 52). These recommendations apply to healthy adolescents and adults and do not include people with abnormal vitamin D or calcium metabolism.

### Limitations.

The limited sample size in our study did not permit separate analyses for participants with very low circulating vitamin D, which has been more strongly associated with our outcomes (19). Additionally, the use of a single recruiting site and modest sample size may limit the generalizability of our results. The average intraassay coefficient of variation (CV) for 1,25(OH)_2_D was 10.4%, while the interassay CV was 17.4%. These relatively high values could raise questions regarding the reliability of the measurements as compared with other variables assessed in the study. Nonetheless, a consistent pattern of decline in 1,25(OH)_2_D concentrations from baseline to 1 week after ACLR was observed in all participants. Furthermore, changes in vitamin D–associated markers may also occur outside of the times when our samples were collected. To our knowledge, our ChIP-seq analysis is the first reported in skeletal muscle; however, the use of a single pooled sample only offers preliminary data of potential VDR targets following ACLR. To confirm the role of VDR in skeletal muscle health after ACLR, pathways that are potentially regulated require additional validation. Lastly, although we were not powered to study modifying effects of skin tone, genetic background, or sex, these factors have the potential to modify vitamin D needs of athletes and warrant dedicated investigation in future studies.

### Conclusion.

Our results demonstrate an elevated level of vitamin D metabolism in quadriceps after ACLR. Utilizing multiomics integration of ChIP-seq and RNA-seq data sets, we have elucidated previously underappreciated pathways through which vitamin D and VDR may regulate human muscle growth after ACLR. Notably, we have observed that having 25(OH)D below 30 ng/mL is associated with a greater reduction in CSA, suggesting that correcting vitamin D status to optimal levels before ACLR could aid in preserving skeletal muscle size during recovery. These findings warrant a randomized clinical trial to evaluate the clinical utility of tailored vitamin D supplementation as a supportive intervention for patients following ACL injury and reconstruction.

## Methods

All participants (*n* = 21) were recruited after an ACL injury and before ACLR surgery, were between 15 and 29 years of age (Table 1), underwent bone-patellar-bone graft ACLR conducted at the University of Kentucky Orthopaedic Surgery & Sports Medicine practice. Thereafter, participants completed a progressive rehabilitation program according to previously published guidelines at the University of Kentucky’s Physical Therapy Department (53, 54). The present study uses outcome data from an observational study (NIH R01AR072061) where participants are enrolled prior to ALCR. The aim of the parent study is to determine whether acute induction of GDF-8 signaling following an ACL injury predicts reductions in muscle strength, connective tissue infiltration, and dysregulation of skeletal muscle progenitor cells. The present manuscript uses CSA, BMD, strength/power measures, and an RNA-seq data set that were collected during the parent study. To maximize the benefit of this observational study, collected samples have been used to answer different research questions (55, 56).

### Circulating biomarkers.

Analyzed serum samples were collected prior to ACLR, and at 1-week, 4-month, and 6-month follow-up visits. Study vitamin D status was defined as the mean 25(OH)D over these 4 time points. Mayo Clinic Laboratories assessed 25(OH)D using gold standard LC-MS/MS methodology. An “optimization” cutoff point was established at 30 ng/mL (75 nmol/L) based on prior literature indicating optimal health outcomes at this concentration (13, 15, 17, 57). ELISA was used to assess 1,25(OH)_2_D (Biovendor, RIS024R and RIS021R), free 25(OH)D (Biovendor, KAPF1991), and DBP (R&D Systems, DY008B and DY3778B-05) according to the manufacturers’ instructions. The average intraassay CV for 1,25(OH)_2_D in our study was 10.4%, while the interassay CV stood at 17.4%. For free 25(OH)D, we observed an average intraassay CV of 5.5% and an interassay CV of 6.6%. For DBP, the average intraassay CV was 5.1%, with an interassay CV of 10.5%.

### Muscle biopsies.

Biopsies were taken from the vastus lateralis on the injured limb and contralateral healthy limb (control) at the time of ACLR and from the injured limb only 1 week and 4 months after ACLR for IHC analysis and protein/gene expression analyses (58). The sample was divided and flash frozen for RNA/protein and for IHC mounted in tragacanth.

### Western blot.

Western blots from muscle biopsies were used to compare VDR and DBP protein before and after ACLR in the injured limb using the healthy limb as a control. Following homogenization, protein concentration was determined with the Bradford assay (Smartspec Plus spectrophotometer, Bio-Rad) to enable us to load 50 μg protein in each well (55). Samples were loaded onto stain-free gels with mouse kidney lysate (VDR positive control) and human VDR-knockout HeLa cell lysate (Abcam, ab257796; VDR negative control). Protein was transferred to a PVDF membrane and probed with VDR antibodies (Abcam, ab109234; 1:1000), and then stripped and blocked before incubating in DBP antibodies (0.25 μg/mL; R&D Systems, DY3778B-05 detection antibody). All blots were analyzed in ImageLab (Bio-Rad) by creating a multichannel image with total protein coupled to the chemiluminescent channel. For each participant, all samples were loaded on the same gel. See complete unedited blots in the supplemental material.

### RNA isolation and RNA-seq.

RNA was isolated from muscle homogenates in accordance with manufacturer guidelines (Direct-zol RNA Miniprep Kit, Zymo). RNA content, purity, and integrity were quantified using the 2100 Bioanalyzer (Agilent) (RIN > 8.5) and the NanoDrop 2000 (Thermo Fisher Scientific) at the University of Kentucky Genomics Core. Six hundred nanograms of total RNA was sent to Novogene Corporation for library construction and sequencing on an Illumina HiSeq 4000 system using a paired-end 150-bp dual-indexing protocol. Raw FASTQ files underwent prealignment quality control, and then were aligned to the GRCh38 reference genome using STAR (https://github.com/alexdobin/STAR). Gene counts were quantified using featureCounts function from the subread package. Differential gene expression was analyzed using DESeq2 (https://bioconductor.org/packages/release/bioc/html/DESeq2.html), excluding genes with maximum read counts of 10 or less. The comparison between ACL-injured samples collected during surgery and 1 week after ACLR were used as input to integrate with the ChIP-seq data. False discovery rate was estimated using the Benjamini-Hochberg step-up method to generate adjusted *P* values. Pathway overrepresentation analysis was performed using g:Profiler (59) with non-ordered query and up- or downregulated genes with adjusted *P* less than 0.05. Gene expression data from human skeletal myocytes were downloaded from the GEO (GSE68323) (32), which included data for cells treated with vehicle or 1,25(OH)_2_D3 with and without VDR knockdown using siRNA (*n* = 4 per group).

### IHC analysis for CSA, MFD, and VDR.

For CSA and MFD analysis, 7-μm sections were rehydrated in PBS and then incubated overnight in a rabbit anti-laminin primary antibody (Sigma-Aldrich, L9393; diluted 1:100 in PBS). Slides were then washed and incubated in Alexa Fluor 555 goat anti-rabbit secondary antibody (Invitrogen, A21429; diluted 1:250 in PBS) for 2 hours, mounted with Vectashield mounting media (Vector Laboratories, H-1000), and imaged on a Ziess AxioImager M2 upright fluorescence microscope. MyoVision, an automated image analysis software, was used to obtain resulting CSA data as previously described (60). For VDR representative images, sections were fixed with 4% paraformaldehyde (PFA) for 7 minutes before antigen retrieval in 10 mM sodium citrate pH 6.5. After cooling, slides were washed in PBS and incubated in 0.5% Triton X-100 in PBS for 5 minutes, washed, and then blocked for 60 minutes in 1% bovine serum albumin. Slides were incubated overnight at room temperature with antibodies against VDR (Santa Cruz Biotechnology, sc-13133; 1:50) and laminin (Sigma-Aldrich, 9393; 1:100). Following a wash, slides were incubated in 3% H_2_O_2_ in PBS for 7 minutes before using a tyramide kit to amplify VDR (Invitrogen, T20913; Alexa Fluor 555) while also using anti-rabbit Alexa Fluor 488 (Invitrogen, A32731) to identify laminin. Following amplification, slides were incubated in DAPI (Invitrogen, D35471) for 10 minutes before mounting and imaging with a Ziess AxioImager M2 upright fluorescence microscope.

### RNAscope.

VDR mRNA spatial distribution in muscle cross sections was assessed with RNAscope in situ hybridization to visualize VDR RNA following the manufacturer’s guidelines (Advanced Cell Diagnostics [ACD]). Briefly, sections were cut at 7-μm thickness and stored at –80°C to preserve mRNA integrity before in situ hybridization. Sections were fixed in 4% PFA for 15 minutes, ethanol dehydrated, and incubated in H_2_O_2_ for 10 minutes to quench endogenous peroxidases. Antigen retrieval was performed using a protease (ACD, 322336). Target mRNA was hybridized with a human VDR probe (ACD, 530961), amplified, and detected using the Opal 570 fluorescent reagent (ACD, 323272). Samples were incubated overnight in anti-laminin primary antibody (Sigma-Aldrich, L9393). Secondary antibody (Thermo Fisher Scientific, A32790) incubation occurred the following day and sections were subsequently DAPI stained.

VDR RNA in situ hybridization images were acquired using a Zeiss LSM 880 upright confocal microscope equipped with an Airyscan detection unit and an argon laser. Imaging was conducted with a 20× (Plan-Apochromat, NA 1.0, water) or a 63× (Plan-Apochromat, NA 1.4, oil) objective lens. DAPI was excited at 405 nm, GFP at 488 nm, and Opal 570 at 561 nm.

### ChIP-seq and bioinformatics analysis.

Skeletal muscle samples were combined for 1-week post-ACLR quadriceps samples from several participants to complete ChIP-seq performed by Active Motif. This approach was necessary to provide the requisite 150 mg of tissue needed for ChIP-seq analysis of VDR in skeletal muscle. We chose not to attempt ChIP-seq with baseline samples because of the overall low abundance of VDR protein in homeostatic skeletal muscle. Immunoprecipitation was achieved using VDR antibody (sc-1008, Santa Cruz Biotechnology), yielding 20 μg of chromatin for profiling. Single-end 75-nt Illumina sequencing reads were mapped to the GRCh38 genome using BWA (https://github.com/lh3/bwa) with default settings after deduplication. Peak calling and motif analysis were completed using MACS2 (61) and HOMER (62), respectively. RNA-seq/ChIP-seq data integration and prediction was done using BETA (31) with basic parameters. The complete sample preparation and data analysis protocol is provided in the Supplemental Methods.

### Strength outcomes.

Participants’ weight-normalized maximum voluntary isometric contraction (MVIC, i.e., peak torque) and the mean slope of the torque-time curve between 20% and 80% of the first 200 milliseconds from muscle contraction onset (RTD_20%–80%_) were evaluated before ACLR and at 4 and 6 month follow-ups using our group’s previously reported protocols (63). Participants completed MVIC and RTD_20%–80%_ testing in both limbs using a Biodex 4 isokinetic dynamometer (Biodex Medical Systems Inc.). Results were analyzed with custom MATLAB code, as previously described (63).

### Bone density measures.

BMD was assessed with dual energy x-ray absorptiometry (DXA) scans (Lunar iDXA, GE Healthcare) and were completed at study baseline and at the 6-month follow-up in both the injured and healthy limbs. We utilized a validated protocol for determining BMD in the femur and tibia (64) in 2 regions, which are outlined in the Supplemental Methods. The DXA enCORE software platform automatically calculated BMD.

### Statistics.

For all outcomes, statistical significance was set at *P* less than 0.05, using 2-sided tests and using adjusted *P* values where appropriate. All continuous measures were summarized with descriptive statistics, and distributions within groups were visually assessed for violations of normality assumptions. To assess relationships between vitamin D status and outcomes (CSA, BMD, and Biodex measures), study 25(OH)D was redefined based on the a priori optimization cutoff value of 30 ng/mL or higher to determine high- (*n* = 8) and low-status groups (*n* = 13). Analyzing vitamin D as a dichotomous variable with a cutoff point of 30 ng/mL is common (15, 65, 66). For each outcome, multiple observations were taken from the same participant over the injured/noninjured legs and across multiple visits. Thus, a full-factorial repeated-measures ANOVA was performed, first analyzing overall differences across the various treatment groups (time point/leg and vitamin D cutoff). Likelihood ratio testing and Akaike Information Criterion (AIC) were used to select an appropriate covariance structure (here, compound symmetry covariance). A Kenward-Roger adjustment was used, as appropriate, to correct for negative bias in the standard errors and degrees of freedom calculations induced by small samples. For each relevant pairwise comparison, estimated differences of means (calculated as Group 1 – Group 2) and the associated standard errors were adjusted for baseline value in the noninjured limb. All available data were analyzed since no observations contained measurement errors and did not show reasons to be removed. All analyses were completed in SAS 9.4 (SAS Institute Inc.).

### Study approval.

All study protocols were approved by the University of Kentucky Institutional Review Board (protocol 43046). All participants provided written and oral consent prior to data collection or parental consent and child assent, where applicable.

### Data availability.

Data for all figures are included in the Supporting Data Values file. RNA-seq data are deposited in the NCBI Gene Expression Omnibus (GEO GSE211681). The ChIP-seq data are available in the GEO under accession GSE243777.

## Author contributions

Experiments were performed in the laboratory of JLF and CSF. JLF, YW, BN, DLJ, and CSF acquired funding and were involved with conception and design of the experiments. DLJ performed all the ACLR procedures. JLF, ANM, CML, BDL, NTT, YW, ARK, and KAR collected and analyzed data. KLT and YW performed study statistics and bioinformatics. JLF and YW drafted the manuscript and created figures. All authors revised and critically edited the manuscript for important intellectual content and approved the final version.

## Supplementary Material

ICMJE disclosure forms

Supporting data values

## Figures and Tables

**Table 1:**
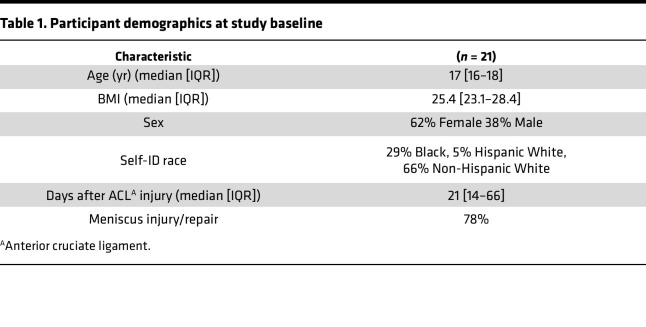
Participant demographics at study baseline

## Version 1 10/19/2023

In-Press Preview

## Version 2 12/08/2023

Electronic publication
